# Effects of hazardous and harmful alcohol use on HIV incidence and sexual behaviour: a cohort study of Kenyan female sex workers

**DOI:** 10.1186/1744-8603-10-22

**Published:** 2014-04-03

**Authors:** Matthew F Chersich, Wilkister Bosire, Nzioki King’ola, Marleen Temmerman, Stanley Luchters

**Affiliations:** 1 Centre for Health Policy, School of Public Health, Faculty of Health Sciences, University of the Witwatersrand, Johannesburg, South Africa; 2 International Centre for Reproductive Health, Department of Obstetrics and Gynaecology, Ghent University, Ghent, Belgium; 3 Wits Reproductive Health and HIV Research Institute, Faculty of Health Sciences, University of the Witwatersrand, Johannesburg, South Africa; 4 International Centre for Reproductive Health, Mombasa, Kenya; 5 School of Public Health, Faculty of Health Sciences, University of the Witwatersrand, Johannesburg, South Africa; 6 Centre for International Health, Burnet Institute, Melbourne, Australia; 7 School of Public Health and Preventive Medicine, Monash University, Melbourne, Australia

**Keywords:** Alcohol, Sub-Saharan Africa, HIV prevention, Cohort study, Kenya, AUDIT test

## Abstract

**Aims:**

To investigate putative links between alcohol use, and unsafe sex and incident HIV infection in sub-Saharan Africa.

**Methods:**

A cohort of 400 HIV-negative female sex workers was established in Mombasa, Kenya. Associations between categories of the Alcohol Use Disorders Identification Test (AUDIT) and the incidence at one year of unsafe sex, HIV and pregnancy were assessed using Cox proportional hazards models. Violence or STIs other than HIV measured at one year was compared across AUDIT categories using multivariate logistic regression.

**Results:**

Participants had high levels of hazardous (17.3%, 69/399) and harmful drinking (9.5%, 38/399), while 36.1% abstained from alcohol. Hazardous and harmful drinkers had more unprotected sex and higher partner numbers than abstainers. Sex while feeling drunk was frequent and associated with lower condom use. Occurrence of condom accidents rose step-wise with each increase in AUDIT category. Compared with non-drinkers, women with harmful drinking had 4.1-fold higher sexual violence (95% CI adjusted odds ratio [AOR] = 1.9-8.9) and 8.4 higher odds of physical violence (95% CI AOR = 3.9-18.0), while hazardous drinkers had 3.1-fold higher physical violence (95% CI AOR = 1.7-5.6). No association was detected between AUDIT category and pregnancy, or infection with Syphilis or *Trichomonas vaginalis*. The adjusted hazard ratio of HIV incidence was 9.6 comparing women with hazardous drinking to non-drinkers (95% CI = 1.1-87.9).

**Conclusions:**

Unsafe sex, partner violence and HIV incidence were higher in women with alcohol use disorders. This prospective study, using validated alcohol measures, indicates that harmful or hazardous alcohol can influence sexual behaviour. Possible mechanisms include increased unprotected sex, condom accidents and exposure to sexual violence. Experimental evidence is required demonstrating that interventions to reduce alcohol use can avert unsafe sex.

## Introduction

**Figure 1:**
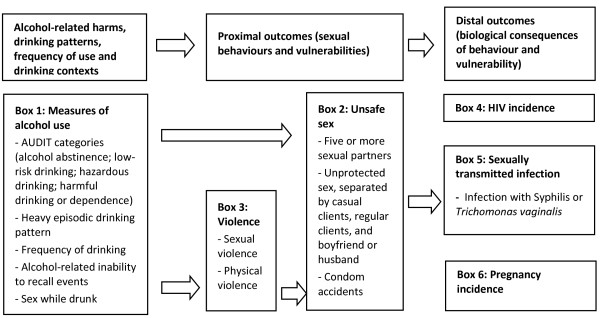
Conceptual framework and study indicators.

When considered separately, harmful alcohol use and unsafe sex account for a considerable portion of disease in eastern and southern Africa [1-3]. There are, however, plausible intersections between these risk factors. In countries with the most severe HIV epidemics in sub-Saharan Africa, such as in Kenya, drinking patterns are characterised by infrequent, but heavy, drinking episodes [4]. Such drinking patterns are most pronounced in the population sub-groups within these countries that have the highest HIV levels [5,6]. Evidence accrued to date, links heavy episodic alcohol use as well as other alcohol use disorders with unsafe sex and HIV transmission (see Conceptual framework for study: Figure 1) [7-9]. Yet, these links have received relatively little research attention and substantial gaps in evidence remain. Alcohol interventions are presently largely ignored within HIV prevention services, with alcohol receiving considerably less attention than interventions for altering sexual behavioural, HIV testing and biomedical factors such as medical male circumcision [10,11]. Similarly, within HIV treatment strategies, harmful drinking reduces adherence to antiretroviral therapy and hastens the disease course of HIV [12-14], but there is little programmatic response or interventional research to date.

Opportunities for alcohol use and sex often co-exist within physical locations, [15-17] and social contexts. In paid sex, both sex workers and clients commonly use alcohol around the time of sexual exchange [7,18-21]. Links between alcohol and unsafe sex may be mediated by alcohol’s pharmacological effects [22]; the nature of drinking venues in Africa [23,24]; intersections between alcohol and other drugs [25]; and also likely by a set of psychological and social influences [26-28]. Consistent and strong associations, including limited evidence of a dose–response effect, [29] have been detected between heavy drinking and incident HIV infection, [11,29] and the proximate determinants of HIV infection, such as multiple partners, unsafe sex and violence [6,29,30]. Meta-analyses in two systematic reviews showed that those who drink alcohol have approximately 70% higher risk for HIV [29,31]. These studies used an array of alcohol exposure measures, and few had longitudinal study designs [31,32].

Sex work is common in sub-Saharan Africa where an estimated 0.7 to 4.3% of women exchange sex for money, goods or favours [33]. A 2012 national household survey in Kenya found that 4% of women had ever received money gifts or favours in exchange for sex, with 1% having done so in the past year [34]. Worldwide sex workers carry a markedly high burden of HIV (almost 13-fold greater than women in the general population) and other sexually transmitted infections (STIs) [35]. Much of HIV risk for these women is a manifestation of their extraordinary social and economic vulnerability [36]. About a third of sex workers in Mombasa Kenya are HIV infected [37]. Also, violence pervades the lives of sex workers in sub-Saharan Africa, with long-term consequences including stress, depression and low self-esteem [38]. Among sex workers in urban and rural Kenya, for examples, about a third reported being raped and 17% had been physically assaulted by a client [39]. Prospective data has demonstrated that effects of gender-based violence on future sexual behaviour are, at least in part, mediated through alcohol use in high-risk contexts in Africa [40]. These linkages are, however, amenable to intervention. A trial in South Africa evaluated a combined counselling and educational intervention, including alcohol reduction, among sex workers and found the intervention group decreased use of substances during sex work, had lower levels of victimisation and fewer STI symptoms than controls [41].

Though there is a growing body of knowledge around the alcohol-HIV nexus, substantial evidence gaps remain. Controversy continues about some components of the alcohol-HIV causal pathway, specifically, it is unknown whether reducing alcohol use would lower risk for HIV transmission. Some contend that associations between alcohol and sexual behaviour are merely due to personality traits (such as sensation seeking), which account for both alcohol use and unsafe sex [11]. Importantly, most studies have not used validated measures of alcohol use to assess these associations [42]. This study, applying validated measures of alcohol use, [6,9] aimed to determine whether alcohol use is associated with unsafe sex, violence and incident HIV infection among sex workers in Kenya. Using validated alcohol indicators wherever possible, associations between study outcomes and several dimensions of alcohol use were examined, such as alcohol harms, patterns of use and alcohol-sex temporality. The study population has considerable variation in alcohol use, facilitating this enquiry. The study intends to contribute additional data to further substantiate the critical need for interventional research on this topic, and to identify which specific characteristics of alcohol use are associated with unsafe sex and violence.

## Methods

### Study setting and design

Sex workers were recruited from their homes, guest houses and the street in two divisions of Mombasa, (Kisauni and Chaani), within the Coast province. This setting was selected as the research team had strong links with the community leaders and sex workers in these divisions, as well as the high incidence of alcohol use, unsafe sex and HIV in the area [37]. A household survey among the general population reported that HIV prevalence in Coast Province was 8.1% in 2007 and 4.3% in 2012 [34]. HIV prevalence among postpartum women attending a large public sector facility in Mombasa was 10.9% in 2006 [43].

The study area was divided into eleven zones, each allocated a field worker. Field workers were familiar with their respective areas and responsible for inviting women to enrol in the study and then maintaining contact with them over the study period. Snowball sampling was used, whereby initial sex workers known to the field worker were invited to participate and those recruited then provided contact details of other sex workers. To be eligible for participation, women had to report receipt of money in exchange for sex as part of their livelihood in the last 6 months, and be at least 16-years old, sexually active in the past 3 months, and not pregnant at the time of enrolment. Women were only eligible if they tested HIV negative with rapid HIV testing at enrolment. Those planning to travel or relocate elsewhere, or who were participating in another HIV intervention study were excluded from participation.

### Study procedures

In this cohort study, women were followed over 12 months with quarterly study visits. Study visits were held at drop-in centres within the community in Kisauni and a local health centre in Chaani. Where indicated, participants received treatment for STIs according to local guidelines or were referred to health services. Contraceptives, including male and female condoms, were offered free of charge. Participants received HIV testing and counselling, and family planning and risk reduction counselling. Women testing HIV positive received post-test counselling and were linked with nearby facilities providing follow-up care and antiretroviral treatment. The Kenyatta National Hospital Ethics and Research Committee approved the study protocol. Participants signed informed consent and provided contact details. Reimbursements of 400 Kenya Shillings were given for travel costs and time taken in interview at baseline and endline (USD6), and 300 Kenya Shillings for other shorter visits (USD4.5).

A structured questionnaire was administered in English or Swahili by a trained research assistant at each of the three-monthly follow-up visits. We collected data on sexual behaviour, pregnancy and HIV status of participants. In addition, at the 12 month visit, data were collected on physical and sexual violence, STIs other than HIV, and alcohol use. A conceptual framework was used to guide selection of study variables and analysis. In this framework, the exposure alcohol use is linked to the proximal outcomes of sexual behaviours and vulnerability. These, in turn, have biological consequences, which were our distal outcomes (HIV, STIs other than HIV and pregnancy).

Study eligibility and HIV status at three monthly follow-up visits were determined using two HIV rapid tests performed in parallel (Uni-Gold™ HIV, Trinity Biotech plc, Bray, Ireland; and Determine™ HIV-1/2, Abbott Laboratories, Abbott Japan Co Ltd, Minato-Ku, Tokyo, Japan). If these results were discordant, an HIV ELISA was done for confirmation. To ascertain that participants were HIV uninfected at enrolment and at study end, pooled RNA real-time polymerase chain reaction (PCR) assays (Generic HIV-1 Charge Virale, Biocentric, Bandol, France) were done [44]. For participants sero-converting during the study, a PCR was done on the last antibody negative blood sample to improve estimation of the timing of infection.

Gynaecological examination and vaginal swabs were done at baseline, after 12 months and at other visits when clinically required. Urine pregnancy tests were performed three monthly. Syphilis infection was detected with a rapid plasma reagin test (Human GmbH, Wiesbaden, Germany). Infection with *Trichomonas vaginalis* was determined by wet mount. Tests were not done for Neisseria gonorrhoeae as previous studies in this population had found a low prevalence of this infection [37,45]. Detection of other STIs, such as herpes simplex virus-2, was not done due to cost and technical constraints.

### Study instruments

Indicators of unsafe sex and violence were used to assess proximal outcomes, hypothesized as intermediate steps between alcohol use and the distal outcomes. Self-reported condom use was dichotomized as ‘always uses condom’ or ‘had unprotected sex’ (no condom use or only intermittently) in the past three months, and separated by casual or regular clients, or with a boyfriend or husband. History of condom breakage, tearing or slippage in the past three months was used as an indicator for condom accidents. Experience of violence from any partner was assessed through a 19-item questionnaire, used by Dunkle et al. [14,16], which drew on the WHO violence against women instrument [17]. To assess sexual violence, women were asked whether any partner had physically forced them to have sex when they did not want to in the past 12 months. Women reporting having been pushed, slapped, hit or kicked by a partner in the past 12 months were classified as having experienced physical violence. Information was collected on six different types of sexual, physical and other forms of violence, whether this had occurred in the past 12 months and, if so, how often (once, few, many times).

Alcohol use, the main study exposure was measured in several ways. The Alcohol Use Disorders Identification Test (AUDIT) [46] was used, and measures of alcohol volume, drinking patterns and temporally-paired alcohol-sex events. A review found that AUDIT has high sensitivity (median 0.83 in 21 studies) and specificity (median 0.87 in 21 studies), across a range of settings and populations, though test validity does vary depending on the AUDIT scores used to define each drinking category [47]. The Cronbach’s Alpha (internal consistency) for the instrument in this population was 0.88. Alcohol exposure was classified as alcohol abstinence (lifetime abstinence or no alcohol use in past 12 months), low-risk drinking (AUDIT score 1–7); hazardous drinking (AUDIT score 8–15), and harmful drinking (AUDIT score ≥16, which includes alcohol dependence) [46]. For analysis, these categories were coded 0, 1, 2 and 3 respectively. Heavy-episodic drinking was defined as five or more drinks on one occasion [48]. Two event-level measures were used to evaluate whether alcohol use and unsafe sex were temporally paired: sex while feeling drunk in the past seven days and condom use while drunk. Frequency of alcohol intake (irrespective of volume per occasion) was categorized as: drinking two to four times a month or less; two to three times a week; or four or more times a week. Total annual alcohol consumption is calculated as the product of number of alcohol units (assuming an alcoholic drink contains 10 g of pure alcohol) and frequency of use [48].

### Data management and analysis

Data were double entered by separate clerks and analyzed using Intercooled Stata 12.1 (Stata Corporation, College Station, TX, USA). Associations were assessed between baseline AUDIT categories, and demographic, sex work and reproductive health characteristics at cohort entry. Chi-square tests were used for analysis of categorical variables, while for continuous variables we used unpaired analysis of variance (variables with a Normal distribution) or Kruskal-Wallis tests (non-Normal variables). The strength of linear associations between continuous variables, for example age at alcohol and sexual debut, was assessed using correlation coefficients (r), derived by simple linear regression.

HIV infection, the primary outcome, was considered a study endpoint for analysis purposes. The time that each woman remained in the cohort and at risk for HIV was summed to give the total person years at risk. Using univariate analysis, we examined associations between AUDIT group and the proximal study outcomes: unsafe sex (partner number and condom use), physical and sexual violence, as well as the distal outcomes (biological measures of pregnancy, HIV and other STIs). Alcohol abstinence constitutes the baseline group in these analyses as no lower level of alcohol consumption that is ‘safe’ for sexual behaviour has been defined, and previous studies suggest a dose–response relationship may exist between alcohol use and unsafe sex [30]. Women’s reported alcohol use at the 12-month interview was used in analysis of associations between AUDIT groups and study outcomes, as these alcohol measures reflect the time period at risk for HIV acquisition and other study outcomes.

Using multivariate models, we investigated whether AUDIT categories were independently associated with the proximal and distal study outcomes, controlling for potential confounding factors. Six Cox proportional hazards models were constructed for outcomes measured at repeated intervals (≥5 sexual partners; unprotected sex with casual client, regular client, or boyfriend or husband; HIV and pregnancy, all assessed at 3, 6, 9 and 12 months). For pregnancy and HIV infection, participants were censored at failure, with the event assumed to occur midway between the corresponding study visits.

Women with laboratory-diagnosed syphilis and/or trichomonas infection at 12 months (STIs other than HIV) were grouped together and compared with women with none of these infections. As STIs other than HIV, and physical and sexual violence were only measured at month 12, we constructed multiple logistic regression models for these binary outcomes. Each model was adjusted for age, religion and level of education, as these variables, considered potential confounders, were associated with the AUDIT categories in univariate analysis.

## Results

Four hundred women were enrolled over a four-month accrual period (May to August 2006) and followed for a year thereafter. A total of 602 women were screened for study eligibility (215 in Chaani and 387 in Kisauni). Exclusion was mainly due to pre-existing HIV-infection (n = 100), refusal of HIV testing (n = 11), pregnancy (n = 20) and declining a gynaecological examination (n = 10). Two women were excluded as they were unable to sign informed consent due to being clearly drunk.

The 400 enrolled participants contributed a total of 381.3 woman-years at risk. Overall, 369 women (92.3%) reached the study endpoint (incident HIV infection) or were retained over the 12-month study period. The mean age of participants was 25.1 years (standard deviation (sd) = 5.2 years) at enrolment, with 22.7% aged 16 to 20 years. About a third of women were Muslim (159/399), a quarter catholic (102/399) and the remaining 40% were mostly protestant religion (154/399). Women had begun sex work on average at 20.4 years with almost a third beginning under 18 years (119/399).

At enrolment, 36.1% of women were classified as alcohol abstainers (144/399; Table 1). Predominately these women reported lifetime abstinence (109/144; 75.7%), and around half were Muslim (46.5%, 67/144). Muslim women constituted about a third of low-risk drinkers (51/148), a quarter of hazardous drinkers (18/69), but only 5.3% of those with harmful alcohol use (2/38). About a quarter were classified as hazardous (69/399; 17.3%) or harmful drinkers (9.5%; 38/399; Box 1 of Figure 1). More than 80% of women with hazardous or harmful alcohol use drank more than once a week (84.1%, 90/107; Table 2). Heavy episodic drinking was also frequent, reported by 71% of harmful drinkers at least once a week. Inability to recall events due to alcohol use was reported by three quarters of harmful drinkers. Women with hazardous or harmful drinking had also commonly used cannabis (15.9%, 17/107), khat^1^ (32.7%, 35/107) or both substances (8.4%, 9/107) in the past week (Table 1). Only four women reported ever using heroin, three of whom used it during the study period.

**Table 1:** Association between alcohol use, and demographic and reproductive health characteristics of female sex workers in Mombasa, Kenya at cohort entry

**Variable**	**Alcohol abstinence n = 144, n (%)**	**Low-risk drinking n = 148, n (%)**	**Hazardous drinking n = 69, n (%)**	**Harmful drinking or dependence n = 38, n (%)**	***P***
**Age** mean years (sd)	24.7 (5.2)	26.1 (5.4)	24.6 (4.9)	24.1 (4.3)	0.04*
**Religion**					
Catholic	23 (16.0)	42 (28.4)	21 (30.4)	16 (42.1)	
Protestant or other	54 (37.5)	55 (37.2)	30 (43.5)	20 (52.6)	
Muslim	67 (46.5)	51 (34.5)	18 (26.1)	2 (5.3)	<0.001
**Highest education level**					
None or primary incomplete	65 (45.1)	67 (45.3)	26 (37.7)	10 (26.3)	
Primary school	45 (31.3)	37 (25.0)	18 (26.1)	11 (29.0)	
Secondary or tertiary level	34 (23.6)	44 (29.7)	25 (36.2)	17 (44.7)	0.13
**Marital status**					
Single	101 (70.1)	101 (68.2)	55 (79.7)	30 (79.0)	
Married or cohabiting	4 (2.8)	6 (4.0)	1 (1.5)	0 (0)	
Separated, divorced or widowed	39 (27.1)	41 (27.7)	13 (18.8)	8 (21.1)	0.48
**Sex work duration** median years (IQR)	3 (2–6)	4 (2–7)	4 (2–8)	3 (2–5)	0.19^
**Part-time sex work**	112 (77.8)	110 (74.3)	50 (72.5)	27 (71.1)	0.76
**Weekly income from sex work**					
≤500 KShs	50 (34.7)	32 (21.9)	10 (14.5)	2 (5.4)	
501-1000	43 (30.0)	48 (32.9)	20 (29.0)	9 (24.3)	
1001-2000	33 (22.9)	41 (28.0)	18 (26.1)	12 (32.4)	
>2000	18 (12.5)	25 (17.1)	21 (30.4)	14 (37.8)	<0.001
**Number of live children**					
0	33 (23.0)	18 (12.2)	16 (23.2)	12 (31.6)	
1	40 (27.8)	59 (39.9)	31 (44.9)	21 (55.3)	
2-3	56 (38.9)	55 (37.2)	20 (29.0)	5 (13.2)	
≥4	15 (10.4)	16 (10.8)	2 (2.9)	0 (0)	<0.001
**Current contraception**					
None	61 (42.4)	42 (28.4)	23 (33.3)	20 (52.6)	
Injectable or implant	44 (30.6)	51 (34.5)	26 (37.7)	8 (21.1)	
Oral contraception	16 (11.1)	18 (12.2)	5 (7.3)	5 (13.2)	
Consistent condoms or other	23 (16.0)	37 (25.0)	15 (21.7)	5 (13.2)	0.25
**Substances other than alcohol**					
Cannabis in past week	1 (0.7)	12 (8.1)	10 (14.5)	7 (18.4)	<0.001
Khat^+^ in past week	12 (8.3)	27 (18.2)	23 (33.3)	12 (31.6)	<0.001

Chi-square test unless indicated; *Anova test; ^Kruskal-Wallis; IQR inter-quartile range; sd standard deviation. KShs Kenya Shillings 1USD = +/-80 KShs; 5 participants had other religion. 15 used contraceptive methods other than those listed. n (%) unless indicated. ^+^Khat, an amphetamine-like stimulant, contains a monoamine alkaloid called cathinone and is a controlled substance in some countries.

**Table 2:** Alcohol volume, drinking patterns and event-level measures among female sex workers in Mombasa, Kenya at cohort entry

**Variable**	**Low-risk drinking (n = 148)**	**Hazardous drinking (n = 69)**	**Harmful drinking or dependence (n = 38)**	***P***
**Age began drinking** mean years (sd)	20.2 (4.3)	18.9 (3.4)	18.2 (4.1)	0.007*
**Annual alcohol consumption** median l/woman (IQR)	2.0 (0.5-4.6)	4.9 (3.2-7.4)	9.4 (7.4-14.6)	0.001^
**Frequency of drinking in past year** n (%)				
2-4 times a month or less	62 (42.2)	15 (21.7)	2 (5.3)	
2-3 times a week	72 (49.0)	43 (62.3)	17 (44.7)	
4 or more times a week	13 (8.8)	11 (15.9)	19 (50.0)	<0.001
**Heavy episodic drinking over past year** n (%)				
None in past year	127 (86.4)	14 (20.6)	3 (7.9)	
Monthly or less	20 (13.6)	35 (51.5)	8 (21.1)	
Weekly or daily	0 (0)	19 (27.9)	27 (71.1)	<0.001
**Alcohol-related inability to recall previous evening in past year** n (%)	4 (2.8)	22 (31.9)	28 (73.7)	<0.001
**Unprotected sex while drunk in past week** n (%)	21 (14.9)	16 (24.2)	13 (35.1)	0.017
**Sex while drunk in past week** n partners (%)				
None	99 (69.2)	35 (50.7)	11 (29.0)	
1-2 partners	33 (23.1)	18 (26.1)	19 (50.0)	
3 or more partners	11 (7.7)	16 (23.2)	8 (21.1)	<0.001

Chi-square test unless indicated; *Anova test; ^Kruskal-Wallis; IQR inter-quartile range; sd standard deviation.

Some women never attended school (7%, 28/399), and a further 35.1% did not complete primary education. Only 8 women had received any tertiary-level training. Education level and religion were associated, with school attendance less common among Muslim women (55.1%, 76/138), than Catholic (39.2%, 40/102) or Protestant women (32.7%, 52/159; *P* < 0.001). About 40% of women with hazardous or harmful drinking had completed secondary or tertiary education (42/107), which was higher than among low-risk drinkers (29.7%, 44/148) and alcohol abstainers (23.6%, 34/144; P = 0.07; Table 1). No association, however, was detected between education level and drinking pattern, when this analysis was stratified by religion.

Approximately 80% of participants had children (320/399), though fewer women with harmful drinking had a child (68%, 26/38; *P* < 0.001) than other groups. Of all women, 36.6% (146/399) were not using contraception at cohort entry, and this was highest among women with harmful drinking, at 52.6% (20/38; *P* = 0.01). Pregnancy occurred in a quarter of women over the study period (25.8%, 103/399), and was 5.3-fold higher among women reporting no condom use than those using injectable contraception or implants (95%CI hazard ratio = 2.9-9.5).

### Association between alcohol use and unsafe sex or violence

Mean age at first sex was 17.2 years (sd = 2.6) and at sex work onset 20.4 years (sd = 4.7), while women began drinking at a mean 19.7 years (sd = 4.1). Age of drinking onset was correlated with age at first sex (r = 0.22, *P* < 0.001). More than half the women had sex before they began alcohol use (59.2%, 170/287), while 29.6% began sex and alcohol use at the same age. Also, age at onset of alcohol use and sex work debut were correlated (r = 0.65, *P* < 0.001). In this instance, alcohol use preceded sex work for 39.1% of women (113/289) and 30.5% started drinking and sex work at the same age.

With each increase in AUDIT category, the median number of partners rose, from 3 (IQR = 2-5) in the past week among alcohol abstainers and low-risk drinkers to 6 partners (IQR = 2-7) in the past week among harmful drinkers (*P* = 0.004; Box 2 of Figure 1). Similarly, multivariate analysis showed that there was a stepwise increase in the likelihood of having five or more partners with each increase in AUDIT category (Table 3). Levels of income from sex work also rose with each increase in AUDIT category, not surprising as number of partners and income levels were associated. Sex workers earning ≤500 Kenya shillings had a median of 3 partners in the past week (IQR = 2-4), compared to a median 5 partners (IQR = 3-7) in those earning >2000 Kenya Shillings.

**Table 3:** Multivariate analysis of associations between hazardous and harmful drinking, and unsafe sex, violence and incident HIV among female sex workers in Mombasa, Kenya

**Outcome variable**	**No. of events**	**Exposure variable**	**Unadjusted ratio(95% CI)**	**Adjusted ratio (95% CI)**	***P***
**Unsafe sex**					
Five or more sexual partners in past 7 days*	218	No drinking	1.0	1.0	
	179	Low-risk drinking	1.18 (0.96-1.43)	1.25 (1.02-1.52)	0.03
	151	Hazardous drinking	1.18 (0.96-1.45)	1.29 (1.04-1.60)	0.02
	129	Harmful or dependant	1.66 (1.33-2.06)	1.70 (1.36-2.13)	<0.001
Unprotected sex with casual clients*	46	No drinking	1.0	1.0	
	32	Low-risk drinking	1.01 (0.64-1.58)	1.03 (0.66-1.62)	0.90
	40	Hazardous drinking	1.49 (0.98-2.28)	1.56 (1.01-2.42)	0.05
	50	Harmful or dependant	3.07 (2.06-4.60)	2.96 (1.95-4.50)	<0.001
Unprotected sex with regular clients*	94	No drinking	1.0	1.0	
	62	Low-risk drinking	0.95 (0.69-1.31)	0.96 (0.69-1.32)	0.76
	81	Hazardous drinking	1.47 (1.09-1.98)	1.52 (1.12-2.07)	0.008
	75	Harmful or dependant	2.25 (1.66-3.05)	2.26 (1.65-3.10)	<0.001
Unprotected sex with boyfriend or husband*	296	No drinking	1.0	1.0	
	215	Low-risk drinking	1.04 (0.87-1.24)	1.05 (0.88-1.26)	0.56
	181	Hazardous drinking	1.04 (0.87-1.25)	1.08 (0.90-1.31)	0.41
	130	Harmful or dependant	1.22 (1.00-1.51)	1.23 (0.99-1.52)	0.06
**Sexual and physical violence**					
**Physically forced to have sex**^**#**^	20	No drinking	1.0	1.0	
	12	Low-risk drinking	0.89 (0.41-1.93)	0.87 (0.40-1.88)	0.72
	19	Hazardous drinking	1.87 (0.93-3.76)	1.75 (0.85-3.58)	0.13
	20	Harmful or dependant	4.21 (2.01-8.82)	4.14 (1.93-8.89)	<0.001
**Physical violence**^**#**^	37	No drinking	1.0	1.0	
	22	Low-risk drinking	0.87 (0.47-1.59)	0.86 (0.47-1.59)	0.61
	41	Hazardous drinking	2.91 (1.64-5.17)	3.07 (1.69-5.58)	<0.001
	36	Harmful or dependant	7.86 (3.76-16.42)	8.40 (3.92-17.96)	<0.001
**Biological outcomes**					
Pregnancy*	39	No drinking	1.0	1.0	
	31	Low-risk drinking	1.16 (0.72-1.85)	1.22 (0.76-1.96)	0.42
	18	Hazardous drinking	0.77 (0.44-1.34)	0.87 (0.49-1.54)	0.63
	15	Harmful or dependant	1.10 (0.61-2.00)	1.02 (0.56-1.88)	0.94
**STI acquisition**^**#**^	7	No drinking	1.0	1.0	
	7	Low-risk drinking	1.49 (0.75-2.94)	1.61 (0.81-3.21)	0.18
	6	Hazardous drinking	0.95 (0.43-2.12)	0.97 (0.43-2.22)	0.96
	4	Harmful or dependant	1.46 (0.63-3.36)	1.43 (0.60-3.39)	0.42
HIV acquisition*	1	No drinking	1.0	1.0	
	2	Low-risk drinking	2.82 (0.26-31.09)	2.66 (0.23-30.81)	0.43
	6	Hazardous drinking	10.5 (1.27-87.58)	9.64 (1.06-87.89)	0.04
	1	Harmful or dependant	2.70 (0.17-43.29)	4.34 (0.24-78.33)	0.32

*Cox proportional hazards regression analysis (hazard ratio); #Logistic regression analysis (odds ratio). All multivariate models adjusted for age, religion and education level. STI is infection with syphilis or trichomonas.

Sex while feeling drunk in the past week was frequent, reported by nearly three quarters of women with harmful drinking (71.1%, 27/38), half of hazardous drinkers (49.3%, 34/69) and even in 30.8% of women with low-risk drinking (44/143; Table 2). A considerable proportion had sex while drunk with three or more partners in the past week (22.4% of women with hazardous or harmful drinking). A third of harmful drinkers had unprotected sex while drunk in the past week, 3.1 fold higher than those with low-risk drinking (95%CI = 1.34-7.17; *P* = 0.005). Also, the proportion reporting condom accidents rose with each increase in AUDIT category, from 11.3% in alcohol abstainers (16/142), 14.9% in low-risk drinkers (14/94), 27.2% in hazardous drinkers (22/81) to 32.7% of women with harmful drinking (16/49; *P* < 0.001).

Multivariate analysis showed that the likelihood of having unprotected sex with clients was higher in women with hazardous or harmful drinking than in alcohol abstainers and low risk drinkers (Table 3). Compared to non-drinkers, the adjusted hazard ratio (AHR) of unprotected sex with a casual client was 1.56 among hazardous drinkers and 2.96 in harmful drinkers. Effect sizes of associations between alcohol use and unprotected sex with a boyfriend or husband were lower than these associations with clients. A trend was noted in this association among harmful drinkers (AHR = 1.23; 95%CI = 0.99-1.52; *P* = 0.06).

Almost a third of women with hazardous or harmful drinking had been physically forced to have sex in the past year (39/130) and 59.2% had experienced physical violence (77/130; Box 3 of Figure 1 and Table 3). Compared with women who abstain, those with harmful drinking were 4-fold more likely to experience sexual violence (adjusted odds ratio (AOR) 95%CI = 1.9-8.9) and 8-fold more likely to experience physical violence (AOR 95%CI = 3.9-18.0). Hazardous drinking was also associated with physical violence (AOR = 3.1; 95%CI = 1.7-5.6).

### Association between alcohol use and biological outcomes

At month 12, 24 women had an STI other than HIV (6.3/100 person years at risk; Table 3). No association was detected between alcohol use and incident pregnancy or STIs other than HIV (Box 5 and 6 of Figure 1). There were 10 incident HIV cases in the cohort (2.6/100 person years at risk). One case occurred in the 142 women with alcohol abstinence over the 12-month period of the study, and seven among the 130 women with hazardous or harmful alcohol use (Box 4 of Figure 1). After adjusting for age, religion and education, the hazard ratio of HIV acquisition was 9.6 fold higher in women with hazardous drinking than those who abstained from alcohol (95%CI = 1.1-87.9; *P* = 0.04); harmful drinking was not associated with HIV acquisition (AHR = 4.3, 95%CI = 0.24-78.3).

## Discussion

Though about a third of the study population did not drink, alcohol-use disorders were common (as assessed with the AUDIT screening tool), as were violence, pregnancy and HIV. Incidence of HIV was higher in women with hazardous drinking than those who abstained from alcohol, and alcohol-use disorders were associated with unsafe sex and violence. The study also demonstrated temporal linkages between alcohol exposure and unsafe sex (unprotected sex while drunk). Links between alcohol and these outcomes might be due to the effects of alcohol on sexual decision-making, condom negotiation and their correct use [26].

Hazardous drinking may form part of social-behavioural interactions that are linked with sex, while alcohol use among people with alcohol harm (such as dependence) is often less social in nature. In people with alcohol dependence, a greater separation of alcohol use and socially-mediated outcomes such as sex, could mean they incur more individual-level harms than social-type outcomes such as unsafe sex [48]. These differences may account for the study demonstrating an association between HIV acquisition and hazardous drinking, but not with harmful drinking. Interestingly, associations between alcohol use and unsafe sex were more apparent among clients than emotional partners. This might reflect the role that alcohol plays in sex work, where it may assist sex workers to approach clients and to cope with sex work [49].

Relationships between drinking and condom accidents, shown in this study, have also been reported previously among sex workers in India [50]. Levels of alcohol use in this study are similar to a previous survey in Mombasa [45] and a longstanding cohort in the city, where 78% of sex workers reported alcohol use [51]. Similarly, almost half of a cohort of sex workers in Nairobi, Kenya reported drinking daily. Compared with other women in that study, those who drank, especially those having three or more drinks a day, used condoms less frequently with clients and had other related HIV risks [52]. In addition, alcohol use among sex workers is substantially higher than among the general population. While about two thirds of sex workers reported current drinking, only 16.6% of urban Kenyans and 11.4% of rural dwellers reported drinking in a national survey [53]. Similarly, sex while drunk was common among sex workers, but only about 5% of youth 15–24 years had sex in the past year when they or their partner was drunk [54].

Complexities inherent in measurements of alcohol, sex and HIV do, however, limit the ability to definitively investigate causality in the absence of trial data. Event-level measures of the alcohol-sex-HIV nexus are difficult to implement as the precise timing of HIV infection is hard to discern. Moreover, gathering alcohol exposure data that is co-terminous with the window periods commonly used for HIV infection is constrained by the need to minimise recall bias with measures of unsafe sex and alcohol. Alcohol itself can hamper recall, with three quarters of harmful drinkers acknowledging that in this study. Employing methods, such as using time-line follow back or providing participants with calendars, may have reduced effects of recall bias in this study.

The relatively small sample size of this study limited the ability to detect associations between some study exposures and outcomes. The study is, however, strengthened by having a relatively large proportion of sex workers who abstained from alcohol and constituted a sizable comparison group. Using the abstinent group as the baseline comparator can be substantiated given the lack of a known lower safe limit of alcohol use for sex. However, use of low-risk drinkers as a baseline group could be considered a more realistic measure of the disease burden which is modifiable (or the standard against which to compare risks of illness), given that low-risk drinking is the major public health goal for most alcohol reduction interventions, rather than complete abstinence [48]. Finally, clearly the ability to generalise these findings to other study populations is limited. Findings may even differ in other sex worker settings, as the study population received a set of interventions as part of the study procedures and benefit from long-standing services in the area [37].

More interventional research on alcohol harm reduction among sex workers is required, examining, for example, the effects of ‘brief interventions’ on unsafe sex in sex work settings [26,49,55]. In addition, applying more complex multilevel interventions would simultaneously aim to shift individual behaviours and the social norms around drinking. One example of a multilevel venue-based intervention was tested in the Philippines, among sex workers in bars, discos, and night clubs [56]. Peer counselling, focused on condom use and sexual negotiation skills, formed the basis for change in individual and social norms. Changes to the bar environment were achieved by working with bar managers to implement HIV prevention practices. The combination of individual, social and environmental interventions appeared to impact on condom use and subsequent STIs. While interventions targeting inter-personal factors and the physical settings within drinking contexts seem promising, the social factors that underlie alcohol-associated HIV-infection risks also warrant attention, such as gender power-imbalances that manifest as alcohol-related gender-based violence [15,16]. While not diminishing the importance of these social factors, given the associations detected between alcohol use and violence, it is important to determine whether interventions to make alcohol use safer can lower women’s experiences of violence.

The findings of this study need to be replicated in further studies, given the small sample and consequent wide confidence intervals. Also, despite the rapidly expanding evidence base and associations documented here, there is insufficient evidence to definitively ascribe the associations observed between alcohol and unsafe sex as being causal in nature [57]. Still lacking is experimental evidence for the reversibility criteria of causality [58]: whether removal or reduction of the putative factor (in this case alcohol) reduces the effect (unsafe sex). We thus cannot discount the possibility that personality traits (such as sensation seeking) or psychiatric disorders account for both alcohol consumption and risky sex, thus creating the associations observed between alcohol and unsafe sex [59,60]. A trial where individual participants are assigned to an experimental and control group, and outcomes such as the presence of semen on vaginal samples are compared between these groups might suffice (assuming that alcohol use is reduced substantially in the experimental arm). Such evidence would provide a more definitive answer to the question of whether heavy alcohol use can alter a person’s decisions about whether to have sex and with whom, as well as whether to use a condom and able to use them correctly. In popular opinion, the influence of heavy drinking on sexual decisions is obvious, and commonly portrayed in the media. The study findings reported here strongly supports the need for a trial that definitively addresses this question. If causal links were demonstrated in such a trial, this would provide the much-needed impetus for alcohol policy reforms in sub-Saharan Africa, and substantially raise the total burden of disease attributed to alcohol from its already high levels. While accruing this evidence is important, interventions are needed now to address alcohol and drug use among sex workers and client groups [61]. Screening for substance use, assigning a risk level and then providing Brief Interventions could markedly improve the health of this population. Validated tools, such as WHO-ASSIST linked to Brief Interventions, take 3–15 minutes to apply and should form part of the services packages being designed for this population [62,63].

## Endnote

^1^Khat, an amphetamine-like stimulant, contains a monoamine alkaloid called cathinone and is a controlled substance in some countries.

## Competing interest

No conflict of interest to declare.

## Authors’ contributions

WB, NK, MT & SL led the design of the study, trained interviewers and coordinated data collection. WB & NK supervised data collection in the field sites. MFC gave input into data collection tools and analysis, helped draft the manuscript, and coordinated overall editing. All authors read, gave substantial input and approved the final manuscript.
